# Nucleoside Analogue Reverse Transcriptase Inhibitors Differentially Inhibit Human LINE-1 Retrotransposition

**DOI:** 10.1371/journal.pone.0001547

**Published:** 2008-02-06

**Authors:** R. Brad Jones, Keith E. Garrison, Jessica C. Wong, Erick H. Duan, Douglas F. Nixon, Mario A. Ostrowski

**Affiliations:** 1 Department of Immunology, University of Toronto, Medical Sciences Building, Toronto, Ontario, Canada; 2 Division of Experimental Medicine, Department of Medicine, University of California San Francisco, San Francisco, California, United States of America; 3 St. Michael's Hospital, Toronto, Canada; Université de Toulouse, France

## Abstract

**Background:**

Intact LINE-1 elements are the only retrotransposons encoded by the human genome known to be capable of autonomous replication. Numerous cases of genetic disease have been traced to gene disruptions caused by LINE-1 retrotransposition events in germ-line cells. In addition, genomic instability resulting from LINE-1 retrotransposition in somatic cells has been proposed as a contributing factor to oncogenesis and to cancer progression. LINE-1 element activity may also play a role in normal physiology.

**Methods and Principal Findings:**

Using an *in vitro* LINE-1 retrotransposition reporter assay, we evaluated the abilities of several antiretroviral compounds to inhibit LINE-1 retrotransposition. The nucleoside analogue reverse transcriptase inhibitors (nRTIs): stavudine, zidovudine, tenofovir disoproxil fumarate, and lamivudine all inhibited LINE-1 retrotransposition with varying degrees of potencies, while the non-nucleoside HIV-1 reverse transcriptase inhibitor nevirapine showed no effect.

**Conclusions/Significance:**

Our data demonstrates the ability for nRTIs to suppress LINE-1 retrotransposition. This is immediately applicable to studies aimed at examining potential roles for LINE-1 retrotransposition in physiological processes. In addition, our data raises novel safety considerations for nRTIs based on their potential to disrupt physiological processes involving LINE-1 retrotransposition.

## Introduction

One of the most striking discoveries resulting from the human genome sequencing project was the observation that our genome is 42% comprised of retrotransposable element (RE) sequence [1]. Long interspersed nuclear element 1 (LINE-1) elements represent the most prolific class of RE, and alone make up 17% of genomic sequences. An estimated 100 retrotransposition competent LINE-1 elements remain in the human genome, of which a small number (6 in the December 2001 freeze of the human genome working draft) are classified as highly active [2], [3]. Intact LINE-1 elements contain two ORFs. ORF1 encodes a 40 kDa protein with RNA chaperone activity, while ORF2 encodes a 150 kDa protein which possesses the endonuclease and reverse transcriptase (RT) activities required for retrotransposition [4]–[13]. Retrotransposition occurs by a mechanism termed target-primed reverse transcription (TPRT) where reverse transcription and integration are coupled as a single concerted step at the site of insertion [14]–[16].

Initial evidence for the presence of retrotransposition competent LINE-1 elements in the human genome was provided by the discovery of LINE-1 insertions into exon 14 of the factor VIII gene in two unrelated haemophilia patients [17]. Many additional cases of genetic disease have since been traced to LINE-1 retrotransposition mediated gene disruptions in the germ-line. These include, amongst others, the insertion of a LINE-1 sequence into intron 5 of the X-linked gene CYBB resulting in aberrant splicing, and manifesting as chronic granulomatous disease, and the insertion of LINE-1 sequence into the 3′ end of exon 44 of the dystrophin gene resulting in a case of Duchenne muscular dystrophy [18]–[21]. LINE-1 retrotransposition in somatic cells has also been reported, and this likely contributes to some cases of carcinogenesis [22]. This is highlighted by the identification of a *de novo* LINE-1 insertion into the APC tumor suppressor gene in colon cancer [23]. Genomic instability induced by LINE-1 retrotransposition may also play a role in the progression of malignancies. A recent study found that LINE-1 promoter hypomethylation, and associated transcription, was significantly more frequent in blast-phase chronic myeloid leukemia (CML) than in chronic-phase CML, and that LINE-1 hypomethylation was prognostic of poorer progression-free survival [24].

While many studies have focused on the role of LINE-1 retrotransposition in pathological conditions, the activity of LINE-1 elements may also play a role in normal physiology. Specifically, it has been suggested that LINE-1 retrotransposition may mediate the generation of neuronal somatic mosaicism during development [25]. Thus there is a two-fold requirement to study pharmacalogical agents with activity against LINE-1 elements. Suppression of LINE-1 elements may provide benefits in cases where their continued activity contributes to pathology, and conversely, inadvertent suppression of LINE-1 elements by agents employed to treat disease states may disrupt LINE-1-mediated physiological processes.

The nucleoside analogue reverse transcriptase inhibitor (nRTI) class of antiviral compounds inhibit a broad range of nucleic acid polymerases including viral RTs, and cellular DNA polymerases. While the prototypical nucleoside analogue, AZT, was originally developed for the treatment of cancer, this class of compounds is now primarily used to treat HIV-1 infection. The safety and utility of nRTIs is dependent upon these compounds having a much greater affinity for viral RT than for cellular DNA polymerases. AZT for example has 100–300 fold greater affinity for HIV-1-RT than for DNA polymerase [26]. Some nRTIs, including 3TC, are also effective at suppressing reverse transcription of the hepatitis B virus [27], [28]. Due to their broad ability to suppress RT enzymes, nRTIs have the potential to suppress LINE-1 retrotransposition. Supporting this, one previous study reported a suppressive effect of the nRTI zidovudine (AZT) on LINE-1 retrotransposition [22].

**Figure 1:**
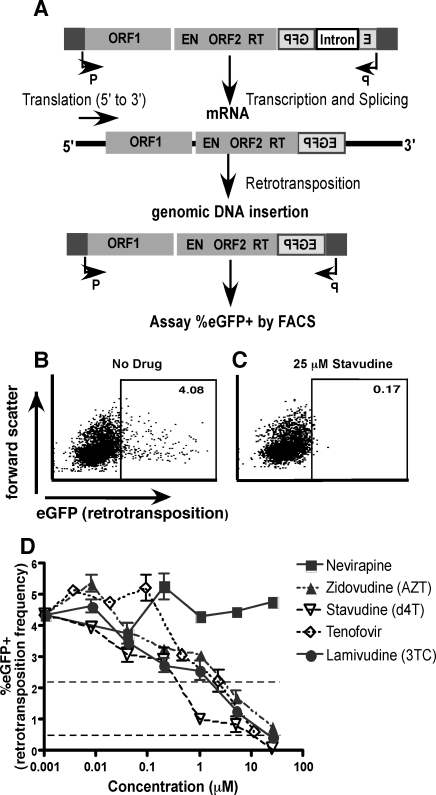
Effect of antiretroviral drugs on LINE-1 retrotransposition frequency. A. The LINE-1 retrotransposition reporter plasmid 99gfpLRE3 encodes the full-length, retrotransposition competent LRE3 LINE-1 element under the control of its natural promoter. An eGFP retrotransposition reporter cassette was inserted into the LRE3 3′ UTR. The cassette encodes eGFP under the control of a CMV promoter, in inverse orientation relative to the LRE3 sequence. The eGFP coding sequence is interrupted by an intron inserted in the same transcriptional orientation as LRE3. Transcription from the CMV promoter does can not yield a spliced eGFP sequence. Transcription from the LINE-1 promoter does not lead to eGFP expression, as the eGFP coding sequence is inverted in the resulting mRNA. However, retrotransposition of this RNA, and integration into the genome, allows a sense eGFP mRNA to be transcribed from the CMV promoter. Thus in cells transfected with 99gfpLRE3, eGFP expression acts as a reporter for the completion of a successful retrotransposition event. The 99gfpJM111 plasmid is analogous to 99gfpLRE3, but incorporates point mutations in ORF1 which render its LINE-1 element retrotransposition incompetent. 99gfpJM111 was therefore employed as a negative control in all assays. Both the 99gfpLRE3 and 99gfpJM111 plasmids also encode puromycin resistance markers allowing for selection of transfected cells. B–D. HeLa cells were incubated in triplicate with five-fold serial dilutions of antiretroviral drugs, and transfected with the LINE-1 retrotransposition reporter plasmid 99gfpLRE3. Transfectants were selected with puromycin. Five days post-transfection, cells were stained with the viability dye 7-AAD, and analyzed by FACS. Retrotransposition frequency was determined by excluding 7-AAD-positive events, and then gating on the eGFP-positive population. Shown is representative data from one of three independent experiments. B. In the absence of drugs, a distinct eGFP-positive population of viable cells, representing cells that have undergone LINE-1-LRE3 retrotransposition events, is clearly distinguishable. Shown is one of six replicates of no drug control. C. In the presence of elevated concentrations of nRTIs, the eGFP-positive population is greatly diminished in frequency, indicating suppression of retrotransposition. Shown is one of three replicates of 25 µM stavudine treatment. D. LINE-1 retrotransposition, as reported by eGFP expression, is inhibited by nRTIs in a dose dependent manner, while nevirapine has no effect. Shown are the mean frequencies of eGFP-positive cells amongst the viable 7-AAD-negative subsets, as determined in triplicate, with standard errors represented by error bars. Dashed horizontal lines indicate 50% and 90% inhibition levels.

We employed an *in vitro* LINE-1 retrotransposition assay to study the effects of nRTIs on LINE-1 retrotransposition. This system is described in detail elsewhere, and portrayed in Figure 1A [29]. Briefly, a retrotransposition competent LINE-1 element (LRE3) was cloned into an expression plasmid, under the control of its natural promoter. An eGFP retrotransposition reporter cassette was then cloned into the 3′ UTR of the LINE-1 element. The cassette consists of an eGFP coding sequence under the control of a CMV promoter inserted in the opposite orientation as the LINE-1 element. The sequence is disrupted by an intron inserted in the same transcriptional orientation as the LINE-1 sequence. Thus transcription from the eGFP CMV promoter yields an unspliced product due to the inversion of the intron in the resultant RNA. Transcription from the LINE-1 promoter yields a spliced transcript, but with an eGFP gene which cannot be translated due to its 3′ to 5′ orientation within the mRNA. Translation of eGFP can only be achieved when this mRNA is integrated into the genome by reverse transcription, allowing sense transcription of spliced eGFP from the CMV promoter. This system is a variation of a neomycin resistance based retrotransposition assay which was developed by Heidmann *et al,* and first applied to studying LINE-1 retrotransposition by Moran *et al* [30] [12].

## Results

The nRTIs studied suppressed LINE-1 retrotransposition with the following hierarchy of potency: stavudine (d4T)>lamivudine (3TC)>tenofovir disoproxil fumarate (TFD)>zidovudine (AZT) (Figure 1B–D). The IC^50^ values for inhibition of LINE-1 retrotransposition were: stavudine–0.22 µM, lamivudine–1.12 µM, tenofovir disoproxil fumarate–1.82 µM, zidovudine–2.21 µM (Table 1). Of the nRTIs tested, only stavudine achieved 90% inhibition of LINE-1 retrotransposition in this experiment, with an IC_90_ of 7.61 µM. As expected, the HIV-1 non-nucleoside analogue reverse transcriptase inhibitor (nnRTI), nevirapine, had no effect on LINE-1 retrotransposition. Nevirapine acts by binding to a hydrophobic pocket adjacent to the catalytic site of HIV-1-RT [31], [32]. Given the dissimilarity of LINE-1 RT and HIV-1 RT, the presence of an analogous binding site in LINE-1-RT was highly unlikely. The HIV-1 integrase inhibitor 118-D-24 also exhibited no suppression of LINE-1 retrotransposition at 5 µM (data not shown).

**Table 1:** 

	LINE-1 IC_50_ (µM)	C_max_ (µM)	Reference
**Stavudine (d4T)**	0.22	2.53 +/− 0.65	34
**Lamivudine (3TC)**	1.12	6.54 +/− 2.18	35
**Tenofovir (TFD)**	1.82	0.47 +/− 0.14	38
**Zidovudine (AZT)**	2.21	4.80 +/− 1.87	36,37

## Discussion

Our study examined the susceptibility of the cloned LINE-1 element LRE3 to nRTIs. Since the estimated 100 copies of intact LINE-1 elements in the human genome represents a substantial degree of diversity, it is important to determine whether the data reported in this study are specific to this particular LINE-1 element, or more globally applicable to the suppression of human LINE-1 retrotransposition. Remarkably, it has been estimated that 84% of the total LINE-1 retrotransposition potential in the human genome can be attributed to 6 ‘hot’ LINE-1 elements [3]. This was determined by cloning 82 intact LINE-1 elements from the genome, and summing their activities using the same *in vitro* retrotransposition assay employed in the current study. In addition, ‘hot’ LINE-1 elements are the progenitors for the majority of disease-causing insertional events isolated *ex vivo* [18]. Brouha *et al* have determined a consensus sequence for these 6 ‘hot’ LINE-1 elements, and demonstrated that a high degree of similarity of a LINE-1 sequence to this consensus is predictive of high-level *in vitro* activity [3]. The LINE-1 element utilized in the current study (LRE3) is one of the most active human LINE-1 elements studied to date. Consistent with this, the amino acid sequence of LRE3 ORF2 is identical to the ‘hot’ LINE-1 element consensus sequence. The data presented here therefore represent an assessment of the susceptibility of the consensus ‘hot’ LINE-1 sequence to nRTIs, and can be reasonably interpreted as a representation of the general susceptibility of human LINE-1 retrotransposition to these drugs.

It is important to note that the data presented in this study cannot be interpreted as an assessment of the general susceptibility of total endogenous reverse transcriptase to these drugs. Indeed, while our data demonstrate a lack of inhibition of LINE-1 retrotransposition by nevirapine, Mangiacasale *et al* have demonstrated an inhibitory effect for nevirapine on total endogenous RT activity [33]. This would suggest that nevirapine is capable of inhibiting other RT enzymes, potentially those encoded by human endogenous retroviruses (HERVs). Homology between the RT enzymes of HIV-1 and HERV-K does allow for the speculation that nevirapine could bind to a hydrophobic pocket in the HERV-K RT enzyme which bears similarity to its HIV-1 counterpart, albeit with lower affinity (10 µM of nevirapine was required to modestly inhibit endogenous RT activity in the study by Mangiacasale *et al*).

LINE-1 retrotransposition has been implicated in the generation of somatic mosaicism of neurons, and other cells over the course of development [25]. If further studies verify roles for LINE-1 retrotransposition in normal physiological processes, our findings may imply an important safety consideration in the future development of nRTIs. Presently, potential nRTIs are evaluated to select candidates with a much greater potency against target pathogen polymerase enzymes than against normal cellular polymerases. Further selection for compounds that are ineffective against LINE-1 RT may reduce the potential for side-effects resulting from inadvertent suppression of LINE-1 mediated physiological processes. One context where suppression of LINE-1 by nRTIs may result in side-effects is in the treatment HIV-1 infection by an antiretroviral (ARV) regimen incorporating an nRTI backbone. Our data support that nRTIs used in the treatment of HIV-1 have the potential to suppress physiological LINE-1 retrotransposition. The IC_50_ values obtained for stavudine, lamivudine, and zidovudine in this study are achieved by standard dosing used in the treatment of HIV-1. Administration of 40 mg BID of stavudine results in a C_max_ of 2.53 +/− 0.65 µM [34], while the C_max_ for 150 mg BID of lamivudine is 6.54 +/− 2.18 µM [35], and the C_max_ for 200 mg zidovudine is 4.80 +/− 1.87 µM [36], [37]. In contrast, the C_max_ for tenofovir disoproxil fumarate at 0.47 +/− 0.14 [38], falls below the LINE-1 retrotransposition IC_50_ value (Table 1). In this regard, stavudine, which was the most potent of the agents tested at inhibiting LINE-1 activity, is associated with the greatest degree of mitochondrial DNA reduction in fat tissue, which is a putative mechanism for lipoatrophy [39]. Also, the French Pediatric Cohort reported on twelve HIV-1 negative children with unexplained neurologic abnormalities who were exposed to nRTIs in utero, and post-partum for the prevention of maternal-fetal HIV-1 transmission [40]. As nRTIs can efficiently pass through the placenta and accumulate in fetal tissues, their potential to lead to such rare neurological defects by disrupting the physiological retrotransposition required for the development of somatic mosaicism, or for other processes, warrants further study [41]–[45].

The potential implication that nRTIs may be of therapeutic use against LINE-1 related diseases is at present a hypothetical interpretation of our data. The genetic diseases that have thus far been clearly attributed to LINE-1 activity result from a past insertional mutagenesis event disrupting a gene. Suppression of further LINE-1 activity will not correct the underlying gene disruption causing the disease. Any therapies stemming from this work will therefore depend on treating disease via suppression of ongoing LINE-1 activity. Further study is required to delineate any causal relationship between LINE-1 promoter hypomethylation and the progression of chronic myeloid leukemia, or other types of malignancies, before inhibition of LINE-1 retrotransposition could be considered as a potential therapeutic. It is also important to note that the retrotransposition assay employed in the current study only detects insertions that are of sufficient length to deliver the entire eGFP coding sequence. The suppression of LINE-1 by nRTIs is expected to occur at the level of reverse transcription. Given the unique TPRT mechanism of LINE-1 retrotransposition, where integration and reverse transcription occur in concert, we cannot rule out the possibility that in the presence of nRTIs the frequency of initiation of retrotransposition remains unchanged, while the length of *de novo* insertions is compromised by inhibition of the RT enzyme. As such short abortive insertions may also contribute to genomic instability this raises an important caveat regarding the utility of nRTIs in a therapeutic setting. Overall, a greater understanding of the potential role of LINE-1 retrotransposition in physiological processes would be required to evaluate the potential risks of therapeutically suppressing LINE-1 retrotransposition.

The more immediate implications of our findings are related to the *in vitro* study of LINE-1 function, where we have demonstrated that nRTIs potently inhibit the generation of new LINE-1 insertions at concentrations that do not significantly impair cellular DNA polymerase enzymes. This provides a proof of principle for using nRTIs in studies aimed at testing the potential role of LINE-1 in physiological processes, by examining the effects of suppressing LINE-1 activity either *in vitro* or in animal models.

## Materials and Methods

### LINE-1 Retrotransposition assay

HeLa cells were plated at 10^5^ cells per well in 6 well plates in DMEM supplemented with 10% FBS (DMEM-10). The following day, media was removed and replaced with DMEM-10 supplemented with either: stavudine, tenofovir disoproxil fumarate, lamivudine, zidovudine, or nevirapine. Stavudine, lamivudine, zidovudine and nevirapine were tested in triplicate at 25 µM, 5 µM, 1 µM, 0.2 µM, 0.04 µM, and 0.008 µΜ. Tenofovir disoproxil fumarate was tested in triplicate at 11.30 µM, 2.26 µM, 0.45 µM, 0.09 µM, and 0.004 µM. No drug was added to 6 wells for controls. Two hours after the addition of drugs a retrotransposition assay was initiated as has been previously described [29]. Briefly, cells were transfected with the LINE-1 retrotransposition reporter plasmid 99gfpLRE3 using FuGene HD (Roche). For each transfection, 3 µl of FuGene HD was added to 100 µl of DMEM (no FBS), and mixed gently. 0.5 µg of 99gfpLRE3 were then added to this solution and complex formation was allowed to proceed for 15 minutes. The full volume of transfection solution was then added to the plated HeLa cells, and these were incubated overnight. Transfection controls were performed in parallel with pEGFP-N1 (Clontech), and efficiencies were determined 48 hour post-transfection by flow-cytometry. We consistently observed 80–90% transfection efficiencies. The following day, and on each subsequent day of the experiment, the medium in each well was replaced with DMEM-10 containing the corresponding concentration of drug, as well as 2.5 µg/ml puromycin. Since the 99gfpLRE3 and 99gfpJM111 plasmids both encode puromycin resistance markers, this allowed for selection of cells that had been successfully transfected. Untransfected controls were also subjected to selection with 2.5 µg/ml puromycin, and consistently were killed within 2–3 days.

On day 5 post-transfection, media was removed from all wells and replaced with 1 ml of 0.5 mM EDTA in PBS. Incubation of cells in this solution for 15 minutes at 37°C resulted in release of HeLa cells from the plate. Cells were transferred to a 96 well plate and stained with the viability dye 7-AAD (BD Pharmingen) following manufacturer's instructions. FACS analysis was performed on unfixed cells using the FACSCalibur system (BD). Retrotransposition frequency was determined by gating on eGFP^+^ cells, after exclusion of dead (7-AAD bright) and apoptotic (7-AAD dim) cells.
